# Association between Related Purine Metabolites and Diabetic Retinopathy in Type 2 Diabetic Patients

**DOI:** 10.1155/2014/651050

**Published:** 2014-02-13

**Authors:** Jianfei Xia, Zonghua Wang, Feifei Zhang

**Affiliations:** College of Chemistry, Chemical Engineering and Environment, Qingdao University, Qingdao 266071, China

## Abstract

*Aims*. The purpose of the study was to investigate the differences of adenosine, adenine, inosine, xanthine, hypoxanthine, and uric acid concentrations in patients with type 2 diabetes mellitus and diabetic retinopathy and assess the relationship between purine metabolites and disease. *Materials and Methods*. The study group consisted of 114 subjects which were divided into three groups: control (*n* = 40), type 2 diabetes without retinopathy (*n* = 35), and type 2 diabetes with retinopathy (*n* = 39). Levels of metabolites were measured in plasma of all participants. *Results*. There is a significant increase of levels of adenosine (0.94 ± 0.17 mg/L versus 0.17 ± 0.01 mg/L, *P* < 0.001), inosine (0.297 ± 0.078 mg/L versus 0.086 ± 0.010 mg/L, *P* < 0.001), xanthine (1.01 ± 0.21 mg/L versus 0.54 ± 0.05 mg/L, *P* = 0.009), and uric acid (70.55 ± 3.97 mg/L versus 53.81 ± 2.36 mg/L, *P* < 0.001) with diabetic retinopathy compared to diabetes mellitus. The levels of adenine, hypoxanthine, and xanthine oxidase did not change. Uric acid, xanthine, inosine, and adenosine correlated positively with systolic blood pressure and urea nitrogen. *Conclusions*. The levels of adenosine, inosine, uric acid, and xanthine may be useful for monitoring the progression of diabetic retinopathy and evaluating the treatment.

## 1. Introduction

As shown by epidemiological studies, the prevalence of type 2 diabetes has a significant rise worldwide, which resulted in an increased burden on individuals and health care systems [1]. For diabetic patients, the development of vascular complications related to microangiopathy and macroangiopathy is one of the most severe problems. In particular, diabetic retinopathy (DR), a kind of serious microvascular complication, is the leading cause of visual impairment in adults aged 30 to 65 years, which draws wide attention for public healthy institution. It is reported that almost all patients develop background retinopathy with time, and 40–50% progress to proliferative retinopathy within 25 years of diabetes onset [2]. Thus, we need effective diagnostic methods and therapeutic tool to prevent the occurrence of DR for diabetic patients. At present, the diagnosis of retinopathy still depended on ophthalmoscopy and fluorescein angiography. However, it is generally acknowledged that only the pathological changes which occurred at the severe stages of the retinopathy can be discovered using this diagnostic method. Therefore, it is of tremendous importance to find out the markers that can be used for the screening and prediction of retinopathy. Several related factors including oxidative stress [3], microalbuminuria [4], gene polymorphisms [5–7], vascular endothelial growth factor receptor-1, thrombospondin-2 [8], and angiopoietin-2 [9] and obesity [10] have been reported to contribute to DR. But diagnosis markers with high precision for DR in individuals with diabetes are still lacking, especially markers that are easy to be measured.

**Figure 1:**
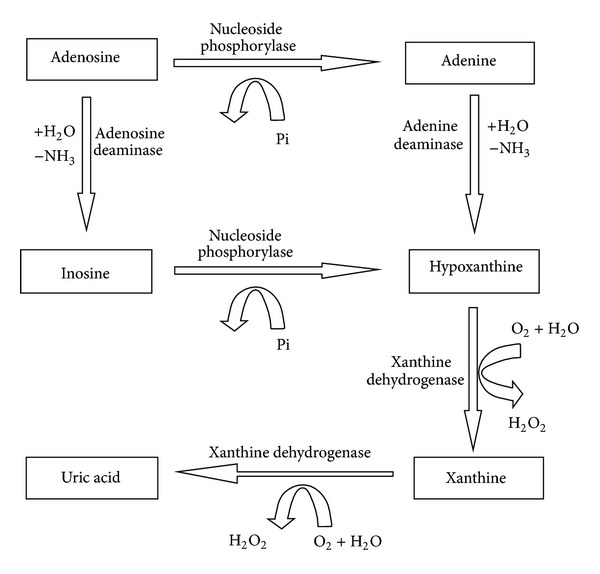
The related purine metabolic pathway.

It is well known that purine metabolic pathway may be strongly associated with the development of diabetic microvascular complication. Adenosine is reported to play an important role in water-electrolyte metabolism, such as retinal blood flow [11, 12], because of its vasoactive property. In purine metabolic cycle, adenosine is phosphorylated into adenine and deaminated rapidly into inosine. Then inosine is converted to hypoxanthine, which is converted to xanthine with the effect of xanthine oxidase. Xanthine also acts as a substrate for xanthine oxidase and enhances superoxide generation [13]. For diabetic patients, superoxide plays a major role in microvascular dysfunction and exerts direct tissue damage, leading to lipid and protein peroxidation. At last, xanthine is metabolized to uric acid, the final product of purine degradation in humans. It was reported that the high level of uric acid was associated with diabetic microvascular complications, such as nephropathy, retinopathy, and neuropathy [14–16], but it is usually considered a marker of tissue dysfunction rather than a risk factor for progression. Recent studies have reported that uric acid might be a true mediator of microvascular and macrovascular disease [17]. Some researchers considered that uric acid might affect the function of vascular smooth muscle cells, which is related to diabetic retinopathy [18]. The metabolic pathway contains these related metabolites and enzymes as depicted in Figure 1. Although the purine metabolism may be associated with diabetic complication, to the best of our knowledge, there has been no detailed report about the relationship between several purine metabolites and DR.

In this paper, we measured six purine metabolites including adenosine, adenine, inosine, xanthine, hypoxanthine, uric acid, and xanthine oxidase (reflected by the uric acid : xanthine) in the plasma of patients with DM and DR. We also observed the relationship between the related metabolites and clinical parameters.

## 2. Materials and Methods

### 2.1. Study Population

The current study included 74 type 2 diabetes patients (41 males and 33 females, age: 56.20 ± 6.22 years) in Beijing, China. Type 2 diabetes was defined in accordance with the criteria of the American Diabetes Association. Their plasma samples were collected as cases. And 40 plasma samples of healthy people (22 males and 18 females, age: 54.41 ± 5.36 years) in the same area were collected as controls. Among these patients, 39 patients had a diagnosis of nonproliferative DR according to International Clinical Diabetic Macular Edema Disease Severity Scale. The DR was determined by ophthalmoscopy and fluorescein angiography through dilated pupils by a retinal specialist prior to the examination of the blood samples, and the patients were classified according to the presence or absence of DR, regardless of its degrees of severity. The other 35 patients were diagnosed to be having type II diabetes without retinopathy by clinician. And the diabetic patients with and without DR were not diagnosed to have nephropathy, neuropathy, or other microvascular or macrovascular complications.

All study participants had given their informed consent, and the Institutional Ethics Committee approved the study. A complete clinical examination was conducted for all subjects. And the related information such as age and duration of diabetes and other details of diabetic therapy were recorded.

### 2.2. Biochemical and Clinical Parameter Analysis

The clinical and biochemical parameters of 74 patients were reviewed and approved by the Clinical Medicinal Research Institute, Sino-Japanese Friendship Hospital, Beijing, China. The diabetic duration was defined as the duration from the first diagnosis of type 2 DM to the time of blood sampling. The dates of first diagnosis of DM2 were obtained from the patients' medical records. Exclusion criteria included acute myocardial infarction, pregnancy, liver disease, stroke, current use of cholesterol lowering agents, and uncertained diabetic duration. Body mass index (BMI) was defined as weight (kg) divided by squared height (m^2^); that is, BMI = W/(H^2^). Blood pressure was taken in the seated position using standardized sphygmomanometers. The clinical and biochemical analyses of all the cases were carried out in 2007 and 2008. Fasting glucose was measured using hexokinase enzymatic reference method. urea nitrogen were measured using diacetyl monoxime method. Hemoglobin A_1c_ (HbA_1c_), and lipid levels were measured using an automatic biochemical analyzer (Hitachi7180, Tokyo, Japan).

### 2.3. Blood Collection and Preparation

Blood samples were collected into EDTA from 6 to 9 o'clock after an overnight fast. All blood samples were centrifuged to obtain plasma in the hospital and sent to our laboratory, where they were stored at −80°C until sample preparation. Before analysis, 800 *μ*L of methanol was added to 200 *μ*L aliquots of plasma, kept in vortex for 2 min, and then centrifuged at 10000 rpm for 15 min at 4°C. The clear supernatant was transferred to a 1.5 mL polypropylene tube and dried under a gentle stream of nitrogen at room temperature. The residue was reconstituted with 100 *μ*L of a mixture of methanol and water (1 : 1, by volume) and stored at 4°C before analysis.

### 2.4. Detection of Related Metabolites in Plasma

Using high-performance liquid chromatography coupled to ultraviolet and tandem mass spectrometry method (HPLC-MS/MS), plasma concentrations of adenosine, adenine, inosine, xanthine, hypoxanthine, and uric acid were simultaneously measured. The HPLC-MS/MS includes an Applied Biosystems (Toronto, Canada) API 3000 triple quadrupole tandem mass spectrometer equipped with a Turbo Ionspray interface for determination and an Agilent 1100 binary HPLC system for separation. Samples were separated on an Agilent TC-C_18_ column with an Alltech guard column. The column temperature was maintained at 25°C, the UV detector was set at 254 nm, and the injection volume was 20 *μ*L. The method has been validated according to the requirement of analytical chemistry [19, 20].

**Figure 2:**
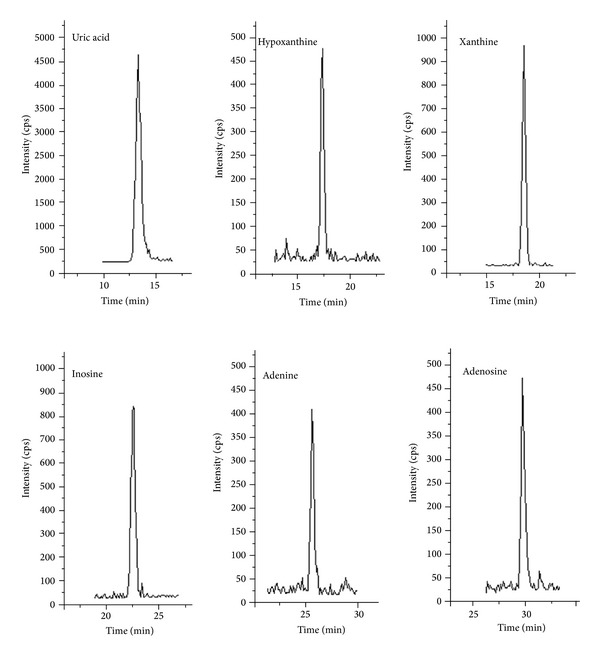
Multiple extracted ion chromatograms of six metabolites in a real plasma sample.

Calibration curves suitable for the analysis of plasma were linear (*r* ^2^ > 0.999) with limits of detection (LOD) from 10 to 50 ng/mL. The recovery for every metabolite is from 79.1% to 102.0% with the relative standard deviation (RSD) less than 10%. Intraday and interday RSD were both lower than 10%.  Figure 2 shows the typical multiple extracted ion chromatograms of these six purine metabolites in a real plasma sample.

### 2.5. Statistical Analysis

All statistical analyses were performed with SPSS statistical package (version 14.0, SPSS Inc., USA). The *χ* ^2^ test and Fisher's exact test were used for the comparison of the clinical characteristics in DM and DR subjects. The data of related clinical parameters and metabolites' concentration were log-transformed to achieve normality. Then the Kolmogorov-Smirnov test was used to verify the normality of the transformed data. The differences between the groups were calculated with Student's *t*-test, whereas linear regression was used to adjust these comparisons for age, duration (years) of DM and BMI. The Spearman or the Pearson correlations were also examined for the research on correlations between metabolites and clinical parameters. In this study, a value of *P* < 0.05 was considered statistically significant. The metabolites, the levels of which have statistically significant differences between two groups of the three groups (Normal, DM, and DR), were defined as potential biomarkers. Receiver Operating Characteristics (ROC) graph was performed.

## 3. Results

### 3.1. Clinical Characteristics of Type 2 DM

A total of 74 patients were enrolled in this study.  Table 1 shows the clinical characteristics of the study groups. From the table, we can see that DM patients with retinopathy (DR patients) have significantly (*P* = 0.034) longer duration of the disease compared to DM patients without retinopathy (DM patients), (13.63 ± 2.08 years versus 10.47 ± 1.48 years). For age, BMI, fasting blood glucose, and HbA_1c_, the differences between the DM patients and DR patients were not statistically significant (*P* = 0.971, 0.481, 0.311, and 0.296). And Lipid profiles such as total cholesterol, triglyceride, high density lipoprotein (HDL), and low density lipoprotein (LDL) of DM patients are not significantly changed compared to DR patients. For blood pressure, systolic blood pressure is significantly (*P* = 0.011) increased in DR patients compared with DM patients (143.07 ± 6.44 mm Hg versus 131.87 ± 4.21 mm Hg) whereas diastolic blood pressure is not changed. In addition, urea nitrogen is significantly (*P* < 0.001) increased in DR patients compared with DM patients (14.71 ± 3.33 mmol/L versus 8.23 ± 1.77 mmol/L).

### 3.2. The Levels of Related Purine Metabolites in Plasma

**Figure 3:**
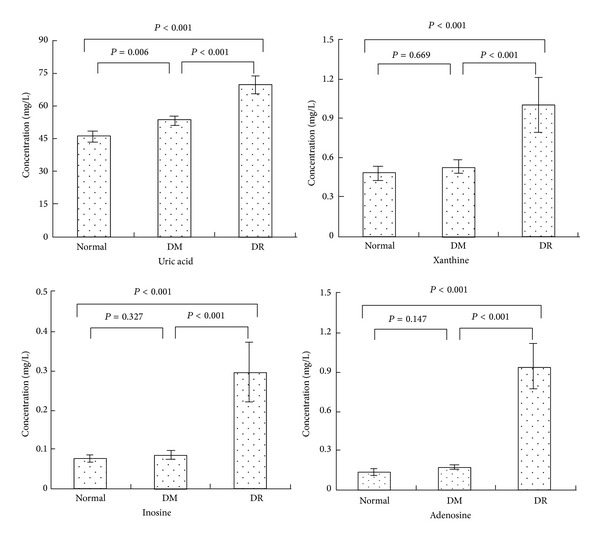
Comparison of four potential biomarkers concentrations in healthy subjects (normal), type 2 diabetes patients without retinopathy (DM), and type 2 diabetes patients with retinopathy (DR). Compared to the group of normal and DM, DR subjects had significantly higher mean plasma concentrations of inosine (*P* < 0.001, *P* < 0.001), adenosine (*P* < 0.001, *P* < 0.001), uric acid (*P* < 0.001, *P* < 0.001), and xanthine (*P* < 0.001, *P* < 0.001). The group of DM had significantly higher mean plasma concentrations of uric acid (*P* = 0.006) compared to the normal group.

The levels of related purine metabolites in plasma were checked. The levels of uric acid and xanthine in the group of DR were significantly higher as compared with DM (70.55 ± 3.97 versus 53.81 ± 2.36, *P* < 0.001; 1.01 ± 0.21 versus 0.54 ± 0.05, *P* = 0.009) and control group (70.55 ± 3.97 versus 46.63 ± 2.41, *P* < 0.001; 1.01 ± 0.21 versus 0.47 ± 0.06, *P* < 0.001). Additionally, also, group of DM had significantly higher level of uric acid as compared to the healthy subjects (53.81 ± 2.36 versus 46.63 ± 2.41, *P* = 0.012). For level of uric acid : xanthine, no statistically significant differences between the groups were observed (Table 2). The levels of inosine and adenosine in the group of DR were significantly higher as compared with DM (0.297 ± 0.078 versus 0.077 ± 0.010, *P* < 0.001; 0.94 ± 0.17 versus 0.17 ± 0.01, *P* < 0.001) and control group (0.297 ± 0.078 versus 0.077 ± 0.010, *P* < 0.001; 0.94 ± 0.17 versus 0.13 ± 0.02, *P* < 0.001). But no statistically significant differences of levels of inosine and adenosine between the group of control and DM were observed. The concentrations of the metabolites with significant changes were shown in Figure 3. For levels of hypoxanthine and adenine, there were no statistically significant differences between any two groups.

### 3.3. Relationship between Correlative Metabolites and Other Parameters

As shown in Table 3, the relationship between levels of metabolites and clinical parameters including age, BMI, systolic blood pressure (SBP), HbA_1c_, and various other variables in all patients of both the DM and DR groups. Uric acid, xanthine, inosine, and adenosine correlated positively with SBP and urea nitrogen. In particular, the Pearson correlation coefficient between any of these four metabolites and urea nitrogen was above 0.568.

### 3.4. The Optimal Numerical Value of Related Metabolite for Predicting the Risk of Retinopathy in Patients with Type 2 Diabetes

**Figure 4:**
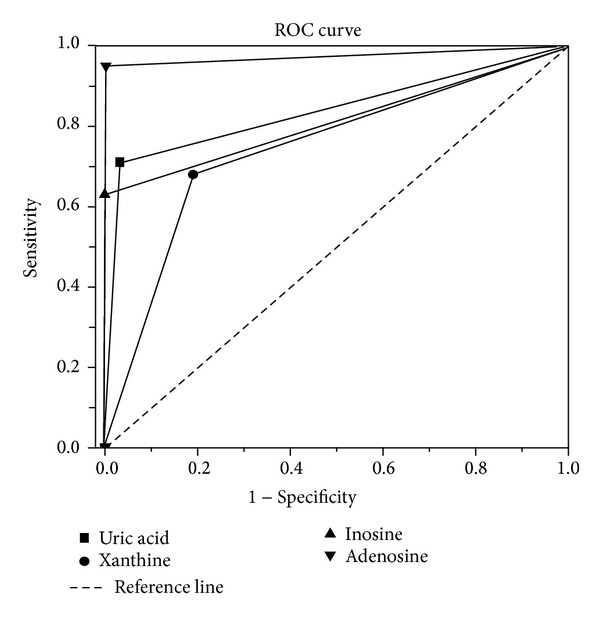
ROC curves of uric acid, xanthine, inosine, and adenosine in groups DM and DR. Adenosine shows more sensitivity and specificity for diabetic retinopathy diagnosis than other potential biomarkers.

ROC curve analysis (uric acid-DR, inosine-DR, xanthine-DE, and adenosine-DR) was used to estimate the optimal numerical value of related metabolites to predict the risk of DR in patients with type 2 diabetes (Figure 4). As a result, the ROC curve of adenosine showed better performance than other potential markers. The area under curve (AUC) of adenosine was 0.913 ± 0.031 and significantly higher than that of null hypothesis (true area was 0.5, *P* < 0.001). This means that the plasma adenosine level could serve as a potential diagnostic marker for DM to DR. Plasma uric acid yielded an AUC of 0.832 with 71.3% sensitivity and 97.2% specificity in discriminating DR. Plasma xanthine yielded AUC of 0.751 with 68.2% sensitivity and 81.3% specificity in discriminating DR. Plasma inosine yielded an AUC of 0.824 with 63.7% sensitivity and 100% specificity in discriminating DR. To find an optimal cutoff point to discriminate dichotomize “diseased” or “healthy”, Youden's index was used in this study. According to Youden's index, the optimal operating point of adenosine was 0.32 mg/L. At this cutoff point, the sensitivity was 94.7% and specificity was 100%. Adenosine = 0.32 mg/L was the optimal numerical value for predicting the risk of DR in patients with type 2 diabetes.

## 4. Discussions

At present, DR remains a major cause of blindness in the world. Many related researches have been carried out worldwide. And the researchers have reported that both genetic and environmental factors determine the susceptibility for the development of DR [21, 22]. It is shown that the development of DR in type 2 diabetes patients was associated with baseline glycemia, glycemic exposure over several years, poor lipid control, higher blood pressure, and smoking [23]. Despite the rapid research progress, robust predictors for the diagnosis of DR in individuals with diabetes are still lacking. Thus, it is necessary to set out the study of biomarker discovery, especially for the low molecular weight metabolites, which are important and easy to be measured. In the present study, four potential biomarkers (adenosine, inosine, xanthine, and uric acid) of retinopathy were shown. And with the ROC curve of these four markers, the plasma adenosine level could serve as a potential diagnostic marker for DM to DR. Adenosine = 0.32 mg/L was the optimal numerical value for predicting the risk of DR in patients with type 2 diabetes (sensitivity was 94.7% and specificity was 100%).

As reported, serum uric acid concentration was found to be independently correlated with insulin resistance [24, 25]. And high level of uric acid is reported to be associated with diabetic complications. Uric acid has some physiologic functions including activation of the rennin angiotensin system and direct actions on endothelial cells and vascular smooth muscle cells. These functions are all related to the occurrence and development of diabetic complications. But in the past researches, uric acid was usually considered as a marker rather than a risk factor for the progression of disease. There is always a controversy that plasma uric acid concentration is a cause or a result of microvascular complications [26]. In this study, we found significantly increased concentration of uric acid in the plasma of DM patients (*P* = 0.011). It demonstrated that the high level of uric acid might be a cause of microvascular complications. In addition, we found that the level of plasma uric acid correlated positively with urea nitrogen (*r* = 0.622, *P* < 0.001) and SBP (*r* = 0.382, *P* < 0.001). There was also a statistically significant difference of the level of plasma uric acid between the group of control and DR (*P* < 0.001), as well as the group of DM and DR (*P* < 0.001). The results demonstrated that uric acid might be a risk factor for DR. In addition, several lines of evidence suggest that increased plasma uric acid may be a significant risk factor of vascular disease. Ioachimescu believes that uric acid may causally/mechanistically contribute to vascular disease [27]. Fukui et al. reported that serum uric acid was associated with microvascular diseases and macrovascular diseases in men with type 2 diabetes mellitus [28]. The evidence is that hyperuricemia stimulated human vascular muscle cells proliferation [29] and increased C-reactive protein expression in these cells. Animal model experiments demonstrate that high level of uric acid in normal rats induced by the uricase inhibitor, oxonic acid, results in hypertension and vascular disease. This led to the hypothesis that uric acid may contribute to progressive vascular disease. And Tseng have reported that increased uric acid concentration is a significant and independent risk factor for peripheral arterial diseases in Taiwanese patients with type 2 DM [30]. Hence, strategies to control and decrease plasma uric acid level may have a beneficial effect on slowing the progression of retinal diseases in clinical practice.

In this study, compared to DM and normal group, the concentration of adenosine in the group of DR was significantly higher with *P* value below 0.001. In addition, adenosine correlated positively with urea nitrogen (*r* = 0.568) and SBP (*r* = 0.336). Therefore, adenosine was supposed to be a potential biomarker for the prediction and diagnosis of diabetic retinopathy, for the guidance and evaluation of treatment. Adenosine plays an important role in water-electrolyte metabolism, such as retinal blood flow and renin release, which make the patients with diabetes be susceptible to retinopathy. For the produce of adenosine, there are two kinds of important enzymes, S-adenosylhomocysteine hydrolase and 5′-nucleotidase. The extracellular metabolism of adenosine is mediated by two mechanisms. First, via an equilibrative facilitated diffusion system, adenosine is taken up quickly and efficiently by red blood cells [31]. Second, adenosine is deaminated rapidly into inosine by adenosine deaminase (ADA), which is found in large amounts particularly in mononuclear cells. And ADA plays a major role in adenosine concentration regulation in both extracellular [32] and intracellular spaces [33, 34]. It is suggested that, with the decrease of the activity and expression of ADA, the production of adenosine increased. Then the elevated adenosine, with the vasoactive property, leads to the generation of superfluous NO. NO, as the important vasoactive substances, affects retinal microcirculation and induces the capillary occlusion, pericytes apoptosis, and matrix membrane thickening, which are closely related to diabetic retinopathy [35].

In this study, the significant difference of xanthine between the group of control and DR (*P* < 0.001), as well as the group of DM and DR (*P* < 0.001), was presented. Therefore, xanthine may be a potential marker for monitoring the progression of DR. Xanthine acts as a substrate for xanthine oxidase and enhances superoxide generation. When xanthine oxidase converts hypoxanthine to xanthine in the presence of molecular oxygen, superoxide radicals (O_2_^−^) are released. Thereby, reactive oxygen species (ROS) is generated, which play a major role in microvascular dysfunction and exert direct tissue damage, leading to lipid peroxidation, denaturation of proteins, and oxidation of DNA [36]. In addition, ROS leads to the increase of vascular endothelial growth factor, which increases vascular permeability and promotes angiogenesis in response to ischemia or hypoxia, resulting in visual impairment. Many direct lines of evidence have demonstrated that ROS was one of the most important mechanisms of DR. But there is also an indefinite viewpoint about xanthine. Previous studies on the effect of xanthine or xanthine oxidase as inducers of microvascular injury have produced inconsistent results. Galat et al. found that infusion of xanthine increased the generation of oxygen free radicals and induced the vascular injury [37]. Another study by Linas et al. found that xanthine oxidase depletion improved vascular function after reperfusion [38]. In this study, the level of xanthine increased with the progression of DR, while the level of xanthine oxidase (reflected by the uric acid : xanthine) did not change. So we suppose that the inducer of vascular injury is not xanthine oxidase but xanthine itself. We also see that there is little change of the level of plasma hypoxanthine while there is an accrescent level of xanthine. Thanks to the invariant activity of xanthine oxidase, the increase of xanthine may come from guanine with the catalysis of guanine deaminase, which catalyzes the hydrolytic deamination of guanine to xanthine.

In summary, plasma adenosine, inosine, uric acid, and xanthine were higher in subjects with DR. Plasma levels of adenosine, inosine, uric acid, and xanthine were also significantly correlated with the urea nitrogen and SBP. The levels of these four metabolites, especially the level of adenosine, may be useful for monitoring the progression of diabetic retinopathy and evaluating the treatment.

## Figures and Tables

**Table 1:** Clinical and biochemical parameters of diabetic patients with retinopathy (DR) and without retinopathy (DM).

Parameters	DM	DR	Statistical significance
*N* (male/female)	35 (20/15)	39 (21/18)	
Age (years)	55.87 ± 7.01	56.52 ± 5.43	*P* = 0.971
Duration of DM (years)	10.47 ± 1.48	13.63 ± 2.08	*P* = 0.034
BMI (kg/m^2^)	25.14 ± 2.63	25.19 ± 3.98	*P* = 0.481
HbA_1c_ (%)	8.13 ± 1.54	8.99 ± 2.03	*P* = 0.296
Fasting blood glucose (mmol/L)	8.45 ± 2.88	7.99 ± 2.11	*P* = 0.311
Triglycerides (mmol/L)	2.27 ± 1.55	2.16 ± 1.25	*P* = 0.318
Total cholesterol (mmol/L)	5.33 ± 0.54	5.13 ± 1.07	*P* = 0.458
HDL (mmol/L)	1.41 ± 0.10	1.25 ± 0.13	*P* = 0.650
LDL (mmol/L)	2.91 ± 0.28	2.96 ± 0.32	*P* = 0.845
Urea nitrogen (mmol/L)	8.23 ± 1.77	14.71 ± 3.33	*P* < 0.001
Systolic blood pressure (mm Hg)	131.87 ± 4.21	143.07 ± 6.44	*P* = 0.011
Diastolic blood pressure (mm Hg)	76.63 ± 2.71	78.97 ± 3.30	*P* = 0.543

**Table 2:** Crude concentrations and adjusted *P* value for six purine metabolites and ratio of uric acid to xanthine in diabetic patients with retinopathy (DR) and without retinopathy (DM) and healthy subjects (normal).

Parameters	Normal (*n* = 41)	DM (*n* = 35)	DR (*n* = 39)	Adjusted *P* value*
Uric acid (mg/L)	46.63 ± 2.41	53.81 ± 2.36	70.55 ± 3.97	*P* = 0.012^a^
				*P* < 0.001^b^
				*P* < 0.001^c^
Hypoxanthine (mg/L)	0.28 ± 0.03	0.26 ± 0.05	0.29 ± 0.06	*P* = 0.474^a^
				*P* = 0.855^b^
				*P* = 0.624^c^
Xanthine (mg/L)	0.47 ± 0.06	0.54 ± 0.05	1.01 ± 0.21	*P* = 0.657^a^
				*P* < 0.001^b^
				*P* = 0.009^c^
Inosine (mg/L)	0.077 ± 0.010	0.086 ± 0.010	0.297 ± 0.078	*P* = 0.368^a^
				*P* < 0.001^b^
				*P* < 0.001^c^
Adenine (mg/L)	0.17 ± 0.03	0.16 ± 0.04	0.16 ± 0.05	*P* = 0.497^a^
				*P* = 0.722^b^
				*P* = 0.654^c^
Adenosine (mg/L)	0.13 ± 0.02	0.17 ± 0.01	0.94 ± 0.17	*P* = 0.137^a^
				*P* < 0.001^b^
				*P* < 0.001^c^
Uric acid : xanthine	99.1 ± 48.71	106.9 ± 12.1	97.1 ± 18.9	*P* = 0.481^a^
				*P* = 0.241^b^
				*P* = 0.225^c^

*Adjusted for age, duration (years) of DM and BMI.

^a^ *P* value from *t*-test between normal and DM.

^b^ *P* value from *t*-test between normal and DR.

^c^ *P* value from *t*-test between DM and DR.

**Table 3:** Correlations between six purine metabolites and clinical parameters.

	Uric acid	Hypoxanthine	Xanthine	Inosine	Adenine	Adenosine
Age	−0.029	−0.037	0.009	−0.043	0.012	−0.080
Duration	0.100	0.171	0.398	0.202	−0.012	0.348
BMI	0.071	−0.065	0.321**	0.206	0.296*	−0.015
HbA_1c_	0.229	0.053	0.171	0.227	−0.005	0.121
Fasting blood glucose	−0.166	−0.125	−0.047	−0.048	0.059	−0.082
Urea nitrogen	0.622**	0.079	0.615**	0.685**	−0.016	0.568**
HDL	−0.016	0.169	0.153	0.027	0.208	0.061
LDL	−0.169	−0.041	−0.021	−0.103	0.011	−0.031
Cholesterol	−0.150	−0.049	−0.075	−0.146	−0.017	−0.129
Triglyceride	−0.063	−0.087	−0.179	−0.138	−0.163	−0.125
SBP	0.382**	0.002	0.453**	0.357**	0.074	0.336**
DBP	0.113	−0.077	−0.003	0.016	−0.029	−0.027

*Correlation is significant at the 0.05 level (2-tailed).

**Correlation is significant at the 0.01 level (2-tailed).
